# EANM/SNMMI practice guideline for [^18^F]FDG PET/CT external beam radiotherapy treatment planning in uterine cervical cancer v1.0

**DOI:** 10.1007/s00259-020-05112-2

**Published:** 2020-12-04

**Authors:** Judit A. Adam, Annika Loft, Cyrus Chargari, Roberto C. Delgado Bolton, Elisabeth Kidd, Heiko Schöder, Patrick Veit-Haibach, Wouter V. Vogel

**Affiliations:** 1 Department of Radiology and Nuclear Medicine, Amsterdam UMC, University of Amsterdam, Amsterdam, Netherlands; 2 Department of Clinical Physiology, Nuclear Medicine and PET, Rigshospitalet, Copenhagen University Hospital, Copenhagen, Denmark; 3 Brachytherapy Unit, Gustave Roussy, Villejuif, France; 4 Institut de Recherche Biomédicale des Armées, Bretigny-sur-Orge, France; 5 French Military Health Academy, Ecole du Val-de-Grâce, Paris, France; 6 Department of Diagnostic Imaging (Radiology) and Nuclear Medicine, San Pedro University Hospital and Centre for Biomedical Research of la Rioja (CIBIR), Logroño, La Rioja Spain; 7 Department of Radiation Oncology, Stanford Cancer Center, Stanford, CA USA; 8 Department of Radiology, Molecular Imaging and Therapy Service, Memorial Sloan Kettering Cancer Center, New York, NY USA; 9 Joint Department of Medical Imaging, University of Toronto, Toronto, Canada; 10 Department of Nuclear Medicine and Radiation Oncology, Netherlands Cancer Institute – Antoni van Leeuwenhoek Hospital, Amsterdam, Netherlands

**Keywords:** Cervical cancer, Positron emission tomography, PET/CT, [^18^F]FDG, Guideline, Radiation oncology, Treatment planning

## Abstract

**Purpose:**

The aim of this EANM / SNMMI Practice Guideline with ESTRO endorsement is to provide general information and specific considerations about [^18^F]FDG PET/CT in advanced uterine cervical cancer for external beam radiotherapy planning with emphasis on staging and target definition, mostly in FIGO stages IB3-IVA and IVB, treated with curative intention.

**Methods:**

Guidelines from related fields, relevant literature and leading experts have been consulted during the development of this guideline. As this field is rapidly evolving, this guideline cannot be seen as definitive, nor is it a summary of all existing protocols. Local variations should be taken into consideration when applying this guideline.

**Conclusion:**

The background, common clinical indications, qualifications and responsibilities of personnel, procedure / specifications of the examination, documentation / reporting and equipment specifications, quality control and radiation safety in imaging is discussed with an emphasis on the multidisciplinary approach.

## Preamble

The Society of Nuclear Medicine and Molecular Imaging (SNMMI) is an international scientific and professional organization founded in 1954 to promote the science, technology, and practical application of nuclear medicine. The European Association of Nuclear Medicine (EANM) is a professional nonprofit medical association that facilitates communication worldwide between individuals pursuing clinical and research excellence in nuclear medicine. The EANM was founded in 1985. SNMMI and EANM members are physicians, technologists, and scientists specializing in the research and practice of nuclear medicine.

The SNMMI and EANM will periodically define new guidelines for nuclear medicine practice to help advance the science of nuclear medicine and to improve the quality of service to patients throughout the world. Existing practice guidelines will be reviewed for revision or renewal, as appropriate, on their fifth anniversary or sooner, if indicated.

Each practice guideline, representing a policy statement by the SNMMI/EANM, has undergone a thorough consensus process in which it has been subjected to extensive review. The SNMMI and EANM recognize that the safe and effective use of diagnostic nuclear medicine imaging requires specific training, skills, and techniques, as described in each document. Reproduction or modification of the published practice guideline by those entities not providing these services is not authorized. These guidelines are an educational tool designed to assist practitioners in providing appropriate care for patients. They are not inflexible rules or requirements of practice and are not intended, nor should they be used, to establish a legal standard of care. For these reasons and those set forth below, both the SNMMI and the EANM caution against the use of these guidelines in litigation in which the clinical decisions of a practitioner are called into question. The ultimate judgment regarding the propriety of any specific procedure or course of action must be made by the physician or medical physicist in light of all the circumstances presented. Thus, there is no implication that an approach differing from the guidelines, standing alone, is below the standard of care. To the contrary, a conscientious practitioner may responsibly adopt a course of action different from that set forth in the guidelines when, in the reasonable judgment of the practitioner, such course of action is indicated by the condition of the patient, limitations of available resources, or advances in knowledge or technology subsequent to publication of the guidelines. The practice of medicine includes both the art and the science of the prevention, diagnosis, alleviation, and treatment of disease. The variety and complexity of human conditions make it impossible to always reach the most appropriate diagnosis or to predict with certainty a particular response to treatment. Therefore, it should be recognized that adherence to these guidelines will not ensure an accurate diagnosis or a successful outcome. All that should be expected is that the practitioner will follow a reasonable course of action based on current knowledge, available resources, and the needs of the patient to deliver effective and safe medical care. The sole purpose of these guidelines is to assist practitioners in achieving this objective.

This guideline is endorsed by the European Society for Radiotherapy and Oncology (ESTRO).

## Introduction

Primary staging of uterine cervical cancer is historically based on non-imaging clinical parameters determined by the FIGO classification [1]. This is mainly due to the fact that cervical cancer is the most common female malignancy in the developing countries where access to imaging facilities is limited. However, in the newest edition of the FIGO classification in 2018 [2], there is allowance of the use of any imaging modality and/or pathological findings for allocating the stage. The FIGO staging system without imaging performs best for microscopic or late-stage disease: clinical and surgical stages correlate in about 90% of cases in patients with stage IA1 disease or stage IIIB and stage IVA disease. For all other stages of disease, this correlation between clinical and surgical stage ranges from 66 to 83% [3].

Imaging should be added to the workup and treatment planning of patients with cervical cancer when available, since it provides significant additional information to determine TNM stage [4] and the best choice of treatment, such as the relationship between primary cancer and adjacent tissue, lymph node involvement, and distant metastases [5]. Furthermore, radiotherapy treatment planning is critically dependent on imaging [6] in order to maximize dose to tumor and spare healthy adjacent tissues. In general, MRI is used for evaluating the local extent of the disease in the pelvis, since it provides excellent soft tissue detail, showing the primary tumor, the relation between the tumor and the adjacent tissues (vagina, rectum, bladder and parametrium), and the involvement of local lymph nodes [7, 8]. The MRI field of view should be expanded with additional abdominal sequence(s) to assess para-aortic node involvement and possible hydronephrosis [1, 3]. When MRI is not available, pelvic-abdominal CT can be performed. Although it is less sensitive and specific compared to MRI in staging of the pelvis, CT can still provide essential information on the involvement of lymph nodes and adjacent tissues, the presence of hydronephrosis, and the distant metastases, compared to clinical staging alone [7]. Although CT remains essential for dosimetry and for imaging of the dose-limiting normal tissue in radiotherapy treatment planning [9], there is development towards MRI-based target definition and treatment planning (such as MR-Linac) [10, 11]. Recent years have also seen a trend towards defining the radiotherapy target volume not just structurally, but also biologically, leading to the term metabolic or biologic target volume (MTV, BTV) [12–14].

Therefore, in addition to anatomical imaging with CT or MRI, molecular imaging with [^18^F]FDG PET/CT is often performed in a clinical setting [15, 16]. For example, in brachytherapy, the ICRU89 report has highlighted this possibility to generate composite clinical gross tumor volume (GTV) by combining available imaging modalities and clinical examination [17]. [^18^F]FDG PET/CT is increasingly becoming a standard part of the imaging workup and treatment planning in patients with advanced uterine cervical cancer who are scheduled to undergo (curative) radio(chemo)therapy, i.e., patients with FIGO stage IB3-IVA or IVB disease due to para-aortic lymph node metastases [18–21]. [^18^F]FDG PET/CT has little value for staging early-stage tumors (FIGO stage IB1 or less), due to its low sensitivity in the detection of small lesions and lymph node metastases in these patients [22, 23].

Although very relevant, there are limited data on the sensitivity and specificity of the different imaging modalities for detecting lymph node metastases in advanced cervical cancer, probably because these patients are treated with (chemo)radiotherapy and histopathological confirmation is not routinely available. Two meta-analyses with high numbers of patients present pooled data for all stages, which is probably an underestimation for advanced disease [24, 25]. In these studies, the sensitivity of MRI for detecting lymph node metastases varies between 0.54 and 0.57 and the specificity between 0.87 and 0.93. For [^18^F]FDG PET/CT, the sensitivity is 0.57, and specificity varies between 0.91 and 0.95. In another recent analysis, the sensitivity for MRI was 0.37–0.71 and specificity 0.83–0.93, while for [^18^F]FDG PET/CT, sensitivity varied between 0.34 and 0.82 and specificity between 0.93 and 1.00 [26]. A recent systematic review showed a prevalence-dependent performance of [^18^F]FDG PET/CT in detecting lymph node metastases in locally advanced cervical cancer. At the highest prevalence, which is the closest representative to the patients treated with radiotherapy, the positive and negative predictive values were 0.96 and 0.81 for pelvic and 0.86 and 0.61 for para-aortic nodes respectively [27].

The following considerations have led to the inclusion of [^18^F]FDG PET/CT in staging, additional to the historical FIGO/TNM staging in locally advanced cervical cancer:

1. Approximately one-half of patients with locally advanced cervical cancer have lymph node metastases at diagnosis. Detection of these nodes is essential for optimal treatment planning [28–30]. [^18^F]FDG PET/CT has a higher accuracy in detection of lymph node metastases (both pelvic and para-aortic) compared to pure anatomical imaging in cervical cancer [31–33]. This can alter the radiotherapy treatment plan, such as extending the radiotherapy field or applying additional radiotherapy dose (boost) to metastatic lymph nodes, in approximately 20% of the patients [34, 35]. Both of these adjustments of the treatment plan have been shown to result in a better survival [36–38].

For groups of patients with the same FIGO stage and treatment, patients with [^18^F]FDG-positive nodes have a significantly worse prognosis compared to those with [^18^F]FDG-negative nodes [39], suggesting that additional treatment of [^18^F]FDG-positive nodes could result in a better survival. The integration of metabolic information gives the possibility to deliver PET-guided concomitant boosts to involved lymph nodes and to potentially improve locoregional control, with acceptable toxicity rates [40].

2. The presence of distant metastases, especially outside the para-aortic area, generally implies a change of treatment regime [5, 41]. As [^18^F]FDG PET/CT scans cover a larger scan region in general (from skull to mid-thigh) compared to MRI (pelvis with alternative extension to abdominal para-aortic), the chance of detecting distant metastases is higher with PET/CT. In particular, in 40% of the patients with suspicious (i.e., [^18^F]FDG-positive) para-aortic nodes, clinically occult supraclavicular nodes are detected on [^18^F]FDG PET/CT as well [42].
3. [^18^F]FDG PET/CT-based target volume delineation reduces the inter-observer variability in radiotherapy treatment planning, as shown for various tumor types [43–45].

Additional aspects, currently under investigation, should also be mentioned:

4. [^18^F]FDG-based metabolic tumor volume (MTV) is a prognostic factor, mainly for highly [^18^F]FDG-avid tumors such as squamous cell carcinoma of the cervix [43]. During treatment, MTV seems to have a role in predicting overall survival [46], similar to the contribution of residual [^18^F]FDG uptake after the completion of treatment [47]. In patients treated with modern radiotherapy modalities, including nodal boosts, the relation between nodal maximal standard uptake value and risk of nodal failure has been shown [48]. Radiomic features extracted from pretreatment [^18^F]FDG PET/CT could also potentially help predicting the risk of local recurrence [49, 50].
5. Dose painting: The imaging of heterogeneity in the metabolic activity [51] or imaging of diverse metabolic pathways within a tumor (such as for glucose metabolism, and hypoxia [15, 52]) allows the use of dose painting [53], the administration of adapted doses for different sub-regions of the tumor. If dose painting is going to benefit the clinical outcome [of (chemo) radiotherapy in uterine cervical cancer], still needs to be elucidated.
6. Including [^18^F]FDG PET/CT in the radiotherapy treatment plan can result in less toxicity to normal organs. It has, for example, been shown that PET-based image-guided IMRT lessens bone marrow toxicity compared to CT-based bone marrow-sparing IMRT in patients with cervical cancer who undergo curative chemoradiation [54].
7. PET/MRI: Based on the theoretical advantages of combining excellent soft tissue detail with metabolic information, integrated PET/MRI has been proposed as a suitable tool for diagnosis and follow-up of cervical cancer [55]. The use of PET/MRI in radiotherapy treatment setting for gynecological tumors still needs to be elucidated in routine clinical practice. Specific aspects, such as MRI-compatible radiotherapy treatment planning equipment (e.g., lasers) and the recognition of radiotherapy attributes (e.g., flat bench) by the attenuation correction software [56], have already been addressed. The remaining challenges include the implementation of multimodal PET/MRI image sets in current CT-based radiotherapy workflows, or the incorporation of PET images in CT-free, MR-Linac-based workflows.

## Goal

The aim of this guideline is to provide general information and specific considerations about [^18^F]FDG PET/CT in advanced uterine cervical cancer for external beam radiotherapy planning with emphasis on staging and target definition, mostly in FIGO stages IB3-IVA and IVB, treated with curative intention.

This field is rapidly evolving, and this guideline cannot be seen as definitive, nor is it a summary of all existing protocols. Local variations should be taken into consideration when applying this guideline, preferably in a multidisciplinary setting.

## Definitions

Members of the EANM Oncology Committee (JA, chair; AL, co-chair; RDB; PVH), the SNMMI Oncology Task Force (HS) and the Advisory Committee on Radiation Oncology Practice (ACROP) of the European Society for Radiotherapy and Oncology (ESTRO) (CC), and invited experts from Europe (WV) and the USA (EK) took part in developing this guideline.

Except the chair and co-chair, authors are listed in alphabetical order. All authors met the non-conflict-of-interest criteria of the EANM/SNMMI/ESTRO.

## Common clinical indications

This guideline describes the practical aspects and special considerations applying to [^18^F]FDG PET/CT in external beam radiotherapy treatment planning in (advanced) uterine cervical cancer.

## Qualifications and responsibilities of personnel

### Physicians

Radiotherapy treatment planning for cervical cancer is at the intersection of radiation oncology, nuclear medicine, and diagnostic radiology. It has been shown that mutual training and close collaboration of specialists from these fields optimize the treatment target delineation process [57, 58]. It appears therefore desirable that treatment planning be approached in a multidisciplinary setting, by professionals trained in multimodality imaging according to local training programs [59], who are also participating in the gynecologic multidisciplinary tumor board.

The target volume delineation and treatment plan are determined by the radiation oncologist. Scan reports generated by a radiologist/nuclear medicine physician should be considered in target definition. In addition, it is recommended to involve the radiologist/nuclear medicine physician directly in the delineation process, depending on the level of experience with [^18^F]FDG PET/CT among radiation oncologists. Where radiation oncology departments own a PET/CT scanner and conduct their own simulation scans, it is required that staff performing the target delineation is properly trained in [^18^F]FDG PET/CT image interpretation. Even in this case, consultation with a radiologist/nuclear medicine physician should be easily accessible, for example, when in doubt of physiology or pathophysiology during the delineation process.

### Technologists

It is necessary that technologists trained in radiotherapy treatment planning are involved in the imaging process. Several scenarios are possible to accommodate this approach, and institutional variations occur according to the established cross training programs. Usually, radiotherapy technologists are responsible for installation of the radiotherapy equipment on the PET/CT (e.g., flat bench and treatment positioning devices), ensuring stable, reproducible, and disease-specific positioning of the patient. The radiotherapy technologist and the nuclear medicine technologist together determine the coverage area for the radiation planning PET/CT and establish the isocenter reference points on the patient. The nuclear medicine technologist is responsible for acquisition of the planning datasets and administering the intravenous (IV) contrast.

In general, all tasks could be executed by the personnel of the department where the scanner is located, if specific knowledge and training has been gained. However, in many cases, collaboration of departments and personnel is required to warrant proper execution of all important aspects. For example, nuclear medicine technologists may prepare patients for optimal biodistribution of [^18^F]FDG, administer the tracer according to radiation safety requirements, and monitor handling of radioactive patients during imaging procedures. Radiation oncology technologists can position patients on the PET/CT scanner according to treatment requirements. Both, the radiation oncology and nuclear medicine technologists will then collaborate to acquire the image datasets and verify the image quality and applicability for treatment planning.

Another option could be that technologists get and maintain special training in each other’s fields to create a pool of technologists available to perform PET/CT in radiotherapy treatment setting.

### Physicists and IT personnel

The multidisciplinary and collaborative approach should apply to the physicists and IT personnel (technical support team) as well. Quality control of the PET/CT should be done by a physicist with special expertise in nuclear medicine. Quality control of the radiotherapy treatment equipment should be done by a physicist with an expertise in radiotherapy [57].

## Procedure/specifications of the examination

As the availability of imaging modalities assisting radiotherapy treatment planning is variable between institutions and continuously evolving, imbedding [^18^F]FDG PET/CT imaging in the radiotherapy treatment plan should be tailored to local workflow. The workflow should be defined and managed in a multidisciplinary manner [57, 60].

### Request

The execution and interpretation of imaging is guided by the clinical questions that need to be answered. The request for a PET/CT in radiotherapy position should be written (preferably digitally) and contain all standard information for an oncological [^18^F]FDG PET/CT. It should explicitly include the request for performing the scan in the radiotherapy treatment position. In most cases, the administration of IV contrast will be requested, and in these cases, kidney function (or eGFR) and history of contrast allergy should be noted.

### Patient preparation and precautions

Patient preparation should be done according to the [^18^F]FDG PET/CT EANM procedural guidelines for tumor imaging version 2.0 [61]. This includes fasting during 6 h prior to imaging, proper hydration, verification of a serum glucose level < 11 mmol/l, and resting in a quiet environment during the [^18^F]FDG uptake time that should ideally last 60 min (± 5 min). The administration of intravenous contrast can contribute to visualization of regional lymph nodes on CT and may also contribute to delineation of the primary tumor. In order to differentiate (pelvic) vessels and lymph nodes, a median portal phase is sufficient (e.g., a 50-s IV contrast delay in a case of a 170-cm patient with a supine, feet-first, skull-base to mid-thigh scan protocol). Administration of oral contrast can be considered in a diluted form (e.g., 5% Telebrix solution) to minimize PET attenuation artifacts. Administering intravenous or diluted oral contrast media does not affect visual assessment of PET/CT in an oncological setting [62–64]. Negative oral contrast (e.g., water) can also been used [65].

Administration of contrast media and premedication should always follow local protocols.

### Radiopharmaceuticals

The administered activity of [^18^F]FDG should follow the EANM/SNMMI guidelines on tumor imaging [61] and should comply with the ALARA principle in the newest generation of scanners, which might allow administration of less [^18^F]FDG [66].

### Hardware

Dedicated PET/CT hardware is required for PET/CT in radiotherapy treatment planning (see section VIII).

### Protocol/image acquisition

In order to maximize the benefits of incorporating metabolic information in treatment planning and to guarantee that the images acquired comply with the requirements for treatment planning and treatment delivery, it is important to be aware of the following factors:

1. Initial patient positioning. Accurate reproducibility of patient positioning is essential when delivering high doses to the tumor, in order to ensure tumor coverage and to protect the surrounding normal tissue, such as the rectum, small bowel, urinary bladder, and pelvic bones [67]; therefore, immobilization devices are routinely used. Patients should be positioned in the PET/CT scanner in the treatment position using a radiation immobilization device on a flat, narrow, and rigid table top for the treatment planning, which should allow registration or indexing of immobilization devices [67]. Immobilization systems must be individualized for each patient and should be anchored to fastening systems, which in turn must be fixed to the treatment table.
2. Accurate alignment. Patient setup should be performed with leveling lasers with lateral and sagittal lasers, to ensure accurate alignment and positioning. The laser light system installed in the PET/CT unit must be in accordance with the one installed in the radiotherapy unit. Quality controls of the laser lights of the PET/CT system must be done routinely to maintain consistency with the treatment unit [67] (see section VIII for quality control as well). Reference ink or tattoo marks of the isocenter should be used (one each on the right side, left side, and ventral center) to ensure reproducibility of setup at the time of treatment [67]. Patient arms, including elbows, should be raised outside of the anticipated treatment field in a comfortable and reproducible position, usually fixed in a device above the head. In case of a sole abdominal scan, holding a ring high on the chest is an option. The [^18^F]FDG PET/CT can be performed for staging and radiotherapy treatment planning in one setting. In this case, the scan may detect unexpected distant metastases, and such patients will not undergo the planned curative radiotherapy treatment, although they may have received unnecessary tattoos prior to the scan. This should be discussed with the patient before the scan. If the same [^18^F]FDG PET/CT is used to perform both staging and radiotherapy treatment planning, intravenous contrast should be administered to ensure proper identification of structures, especially lymph nodes, unless contra-indicated.
3. Combination of procedures. An alternative strategy is to perform [^18^F]FDG PET with low dose CT and subsequently co-register the images with a separately acquired planning CT. In this approach, it needs to be ensured that the [^18^F]FDG PET/CT scan still adheres to the described requirements for patient positioning and that image registration is performed with the highest possible accuracy and quality control. In case of co-registrations, registration errors can occur, so a protocol for checking these registrations should be in place.
4. Scan region and direction. Since the pelvis is the area of focus, performing the PET scan in the caudal to cranial direction can help reduce artifacts of bladder filling and bowel peristalsis [68]. Pelvic organs physiologically change their positions according to the fullness of the bladder, rectum, or bowels. Therefore, movement of the cervix and uterus due to bladder/bowel filling needs to be taken into account during radiation treatment planning, especially with intensity-modulated radiation (IMRT) [69, 70].
5. Management and evaluation of bladder filling. Bladder filling is a critical issue, as it may vary from planning (PET/)CT to treatment, and during treatment, from one fraction to another. Up to date, there is no consensus on what constitutes the best strategy to deal with this changing anatomy. The definitive imaging protocol should be developed in collaboration between departments taking all available imaging modalities into account. Limitations related to bladder filling should be considered when integrating primary staging PET/CT findings into treatment planning.

In general, priority should be given to sensitivity and specificity when the exam is performed as part of primary staging. Therefore, acquisition should be performed with an empty bladder. Patients should void just prior to the [^18^F]FDG PET/CT [71]. In most cases, this is sufficient to ensure that proper interpretation of the scan and extra intervention is not necessary. Alternatively, patients can have a Foley catheter placed prior to the [^18^F]FDG injection, and then following the [^18^F]FDG injection, 20–40 mg (0.5 mg/kg body weight) of furosemide can be administered intravenously along with continued normal saline aiming to give approximately 1 l of i.v. fluid [72, 73]. It is important that the Foley catheter be placed to gravity, below the patient to allow emptying of the bladder. This approach can potentially decrease the amount of [^18^F]FDG in the ureters as well as the bladder. In rare cases, it may be difficult to distinguish between local [^18^F]FDG activity in ureters and small PET positive lymph nodes, and an additional limited scan after voiding could be helpful. Bladder irrigation is mainly used in diagnosing bladder cancer and is not necessary in this setting [74].

When [^18^F]FDG PET/CT is used for target volume delineation, the following options are possible:

1. Comfortably filled bladder on the treatment planning CT and throughout the treatment. Drinking protocols are recommended to achieve this, with specifications on timing of voiding and timing and volume of fluid intake, in an attempt to have treatment as reproducible as possible [75]. Performing PET/CT with comfortably filled bladder would be ideal for bony fusion with treatment planning CT to guide tumor target delineation, but it can be suboptimal for proper interpretation of the PET/CT images because of physiological [^18^F]FDG activity in the bladder.
2. Full and empty bladder scans at the time of treatment planning provide information about the range of internal motion of the target volumes. Performing PET/CT with empty bladder renders bony fusion hazardous but improves PET/CT interpretation by minimizing the amount of activity in the bladder. Availability of scans in both configurations provides information about the range of internal motion of the target volumes to generate an internal target volume (ITV) with individualized margins.
3. Another approach considering bladder movements is fusion of diagnostic and treatment planning imaging series, including PET/CT, with different situations of bladder filling. These sets of scans with different anatomical situations can be used to generate a tailored ITV for the cervix and uterus region, as part of an optimization process of contouring protocols. Such complex contouring protocols based on multiple imaging series available with different combinations of bladder filling are currently being tested and evaluated prospectively in a multicenter setting in the EMBRACE II study [76].

### Interpretation/target volume delineation

The gross target volume (GTV) of the primary tumor and pathological lymph nodes are usually defined on MRI (T2 imaging), supported by gynecological examination. The metabolic tumor volume (MTV), defined as tissue with pathological [^18^F]FDG uptake, is an essential part of the total volume that needs to be treated. It identifies macroscopic tumor locations, with biological characteristics that are thought to negatively affect prognosis and response to treatment and thus require inclusion in a GTV or boost area [77]. The goal is to maximize disease control of the primary tumor and nodal metastasis alike. For this purpose, [^18^F]FDG PET/CT is generally assessed using visual criteria in the appropriate clinical context. The limited spatial resolution and the “natural blurring” of the PET images mean that delineation on PET alone can be challenging. Delineation of the primary tumor and lymph nodes is primarily based on anatomical information provided by CT and/or MRI, taking into account the findings from gynecological examination, while [^18^F]FDG PET/CT is mostly used for additional identification and localization of suspicious lymph nodes and detection of distant metastases.

Non-physiologic [^18^F]FDG accumulation on PET images should be interpreted as pathological, especially when focal, with additional consideration of signal intensity [61]. The identification of abnormal uptake is affected by the contrast between the tumor and its surroundings. This contrast is related to several pathophysiological factors, the most significant of which are lesion size and histology ([^18^F]FDG avidity of the tumor), volume of vital tumor cells, patient movement during image acquisition, and physiological high uptake in adjacent background [61]. This also translates to strategies to derive a contour for target definition: The border of a target volume should be positioned to enclose the metabolic tumor volume considering these factors. All available information, such as the results of the additional anatomical imaging, should be considered when defining the definitive target volume.

The primary tumor should be histologically verified before the start of any (curative) treatment. When interpreting the PET/CT in uterine cervical cancer, the histological subtype of the tumor should be taken into account (e.g., squamous cell carcinoma is highly [^18^F]FDG avid, whereas mucinous adenocarcinoma often shows low [^18^F]FDG uptake) [78]. Only tumors that are sufficiently [^18^F]FDG avid can be staged properly with [^18^F]FDG PET/CT, and reduced sensitivity for local tumor extension and metastatic disease must be taken into account in case of less [^18^F]FDG-avid tumors.

Certain standard criteria for lymph node evaluation in malignancies also apply to uterine cervical cancer [79]. In general, lymph nodes with short axis larger than 1 cm, any lymph node with central necrosis, high IV contrast media uptake, loss of fatty hilum, or signs of extra-capsular spread should be considered pathological on anatomical images [80]. Regardless of these criteria, corresponding [^18^F]FDG activity higher than in normal surrounding tissue is suspicious for metastasis [61]. In general, all suspicious nodes should be included in the radiotherapy treatment plan. However, some reactive nodes may also show [^18^F]FDG uptake. Therefore, PET findings should be put into clinical perspective, and treatment options should be discussed in the multidisciplinary tumor board considering the known lymphatic drainage patterns in gynecological cancers: For instance, a small but [^18^F]FDG-avid node in a typical nodal basin or in the vicinity of other clearly involved nodes should be considered malignant, while a similar node in an aberrant location may be ignored or considered for verification. Moreover, large nodes with massive central necrosis and only a small rim of remaining nodal tissue may show very little [^18^F]FDG uptake, leading to a false-negative signal.

The diagnostic performance of PET/CT for defining pathological para-aortic lymph nodes is high with sensitivity of 83% and specificity of 91% [25]. However, there is a possibility of false-negative para-aortic nodes on [^18^F]FDG PET/CT, reported in up to 22% of those with pelvic nodal metastases [81–83]. Therefore, para-aortic lymph node dissection prior to radiotherapy in patients with pelvic and without para-aortal metastases on PET/CT could be considered.

When [^18^F]FDG PET/CT is used to assist in delineation of macroscopic tumor, interpretation of the images is generally visual and supported by anatomical imaging. However, visual interpretation and manual contouring of multimodal image data are subject to observer variation. Auto-contouring involves algorithm-based methods to derive tumor borders from metabolic information on PET/CT. However, this contour may not be perfect given the limited spatial resolution of PET as well as inter- and intra-tumoral biological variations and inhomogeneity. In addition, clinical MR imaging findings also need to be considered for generating an adequate GTV. As such, auto-contours require adjustments; they may assist, but cannot replace, the visual interpretation by trained observers. Modifications accounting for bladder filling status, discussed above, are also required. However, one major advantage of auto-contouring of tumor volumes may be improved interobserver agreement [84]. There are many different auto-contouring algorithms available; it is currently unknown which performs best in the setting of cervical cancer [85]. As previously described, a simple threshold of 40% of SUV_max_ can be sufficient for automatic tumor delineation on [^18^F]FDG PET/CT in highly FDG-avid cervical cancer [86], but this does not eliminate the need for subsequent visual verification and manual optimization and adjustment for clinical MR imaging findings. Especially in less [^18^F]FDG-avid tumors, manual adjustment of the tumor volume is usually necessary, in particular to exclude excreted [^18^F]FDG in the urinary bladder. In tumors with only mild [^18^F]FDG uptake and/or small volume, the delineation of the primary tumor and involved lymph nodes on PET/CT can be challenging. In these cases, anatomical imaging, preferably MRI, should serve as the primary imaging basis for RT treatment planning.

Knowledge of pathological and physiological [^18^F]FDG uptake is essential for the interpretation of PET images and in the delineation process, since several pelvic organs may show variable physiological [^18^F]FDG uptake (e.g., the ovaries, endometrium, ureters, and urinary bladder) [87]. Variable [^18^F]FDG uptake in ovaries during the menstrual cycle and the differences in physiologic [^18^F]FDG uptake patterns between pre- and postmenopausal women should be taken into account [88]. Presence of distant metastases should always be discussed in the tumor board, as this is likely to change the treatment plan from curative to palliative.

In some instances, a surgical dissection of bulky nodes (mostly > 2.5 cm short axis) is performed to optimize the effect of subsequent radiotherapy [89]. If the PET/CT is performed shortly thereafter, there is an increased chance of false-positive findings (i.e., reactive nodes) [90]. Postsurgical lymphoceles and/or pertinent surgical clips should be included in the clinical target volume (CTV) delineation field [91].

When the PET/CT is not acquired in the treatment position, a visual correlation between the planning CT and the PET/CT can be made, and the metabolic information can be included in the target volume delineation. Although this approach is obviously less accurate than performing the PET/CT in the treatment position, significant additional information can still be retrieved from that PET/CT compared to anatomical imaging, especially with regard to lymph node involvement [7, 32, 92].

Visual correlation between the MRI and the PET/CT could be challenging, for example, when MR images for uterine cervical cancer are acquired perpendicularly to the long axis of the cervical canal, while the PET/CT is acquired without angulation [8].

## Documentation/reporting

The [^18^F]FDG PET/CT scans should preferably be reported by a nuclear medicine physician and/or a radiologist trained in [^18^F]FDG PET/CT image interpretation with experience in gynecological malignancies. Depending on the local circumstances and national re-imbursement plans, one joint report for the CT and PET portions or two separate reports can be issued. If two separate reports are issued, a brief integrated summary of key findings should be added to one of these reports.

The report should contain the main clinical information (with a separate entry of additional clinical data gained from the patient chart or by consultation of the referring physician), the clinical question, and technical details, including the administered [^18^F]FDG activity; the serum glucose level prior to administration of [^18^F]FDG; the site of [^18^F]FDG administration; the [^18^F]FDG uptake time; the field of view of the scan; the CT protocol (low dose or dedicated); the additional series that were acquired, if applicable (e.g., pelvis, full bladder, prone); the details on administered i.v., oral or vaginal contrast (including amount and brand name); any pre-medications (generic name and amount); and the fact that the PET/CT was performed in the radiotherapy setting.

The report should also mention any imaging studies used for comparison and correlation, with type of scan and date.

We encourage the use of a standardized report template, with clear entries for the various body regions and organs and an enumerated conclusion with recommendation of suggested additional/follow-up imaging, if applicable.

When the PET/CT scans are directly used for radiotherapy target delineation, the person performing the delineation should be trained in [^18^F]FDG PET/CT image interpretation (see section V) [93].

## Equipment specifications, quality control, and radiation safety in imaging

The EANM procedural guideline for tumor imaging applies for this section [61]. The PET/CT equipment used for radiotherapy treatment should comply with additional hardware requirements for radiotherapy treatment planning, such as a flat table top, positioning aids and devices fixed to the flat table top, planning laser systems, and increased gantry diameter if possible [57].

The quality control (QC) of the PET/CT hardware should follow national/international guidelines [57] and should include the QC of the CT [94, 95], the PET [96], and the PET-CT alignment. There are no radiotherapy-specific PET/CT QC guidelines yet. QC steps according to radiotherapy recommendations should be followed, including positioning and movement of table under constant load, artifacts of table top, and laser geometry and accuracy [97].

The radiation burden from imaging has to be put into perspective in case of patients receiving external beam radiotherapy and in our opinion is negligible in this setting.

Historically, almost half of the radiation exposure of technologists is related to patient positioning [98]. Several measures can be taken to limit exposure to personnel, such as sufficient patient instructions prior to administration of [^18^F]FDG, trained staff to shorten positioning time, and room preparation prior to patient arrival [66, 99].

## Safety, infection control, and patient education concerns

Local hospital safety protocols should be followed in any case.

## Abbreviations

ALARA: As low as reasonably achievable
BTV: Biological target volume
CT: Computed tomography
EANM: European Association of Nuclear Medicine
eGFR: Estimated glomerular filtration rate
ESTRO: European Society for Radiotherapy and Oncology
[^18^F]FDG: 2-deoxy-2-[^18^F]fluoro-D-glucose
FIGO: International Federation of Gynecology and Obstetrics
GTV: Gross tumor volume
IG-IMRT: Image-guided intensity-modulated radiation therapy
IMRT: Intensity-modulated radiation therapy
i.v.: Intravenous
MTV: Metabolic tumor volume
MRI: Magnetic resonance imaging
MR-Linac: MRI-guided radiotherapy
PET-CT: Positron emission tomography computed tomography
RT: Radiotherapy
SNMMI: Society of Nuclear Medicine and Molecular Imaging
SUV: Standard uptake value
SUV_max_: Maximal standardized uptake value
TVD: Target volume delineation
